# A Case Report of Schizophrenia With a Partially Empty Sella: Related or Incidental?

**DOI:** 10.7759/cureus.65322

**Published:** 2024-07-25

**Authors:** Anshita Girdhar, Pradeep S Patil

**Affiliations:** 1 Psychiatry, Jawaharlal Nehru Medical College, Datta Meghe Institute of Higher Education and Research, Wardha, IND

**Keywords:** neurobiology, psychiatric illness exacerbation, magnetic resonance imaging, partially empty sella, schizophrenia

## Abstract

A 55-year-old male patient who had a history of schizophrenia for 20 years was admitted to the Department of Psychiatry, in a tertiary care center. The patient presented with an exacerbation of symptoms for three months, with no apparent cause, leading to the suspicion of an underlying organic cause. The patient was overweight, was newly diagnosed as a case of type 2 diabetes mellitus, and also had swelling over his left ear. The magnetic resonance imaging (MRI) brain and laboratory testing that were done while he was in the hospital revealed the existence of a partially empty sella. With the MRI findings, it can be said that the partially empty sella and the occurrence of schizophrenia can be related to each other. In the existing literature, a few studies indicate the correlation between the two, and future studies can help with identifying the association.

## Introduction

Schizophrenia is a complicated psychiatric condition with multifaceted causative factors and proven genetic predisposition. Epigenetic processes, environmental effects, and numerous susceptibility genes interact to determine its expression [1]. With a lifetime frequency of around 1% in the general population, schizophrenia is a complex, diverse psychiatric condition impacted by both hereditary and environmental variables. This illness presents with a range of symptoms, including (i) positive symptoms, like hallucinations, delusions, thought disorders, as well as psychomotor agitation; (ii) negative symptoms, like emotional blunting, social disengagement, and deficiencies in motivation and reward processing; and (iii) cognitive impairment, which impacts memory, working memory, learning, attention, and executive function. Mood disorders and anxiety disorders are also prevalent among patients with schizophrenia [2].

It is well known that there is a higher risk for schizophrenia in the relatives of schizophrenic patients than in the general population [3]. Twin studies and adoption studies indicate that along with genetic factors, environmental factors also play a major role in the familial transmission of schizophrenia [4,5]. A variety of twins and triplets were analyzed through genetic studies, to establish genetic predisposition to psychotic spectrum of diseases. As a result, the need has arisen to look for cues of early diagnosis in established investigations in search for specific endophenotypes of schizophrenia. MRI brain scan incidental findings have thus gained much role in need for interpretation for early detection and predisposition to psychotic illnesses. Previous studies in monozygotic twins and triplets suffering from schizophrenia are characterized by great similarities in MRI findings such as borderline ventricular enlargement, widened subarachnoid spaces [6]. There are many similarities in the symptomatology, illness course, response to neuroleptic medication treatment, and findings related to neuropsychological assessment, such as enlarged subarachnoid spaces, empty sella, and borderline ventricular enlargement. The MRI assessment can be used to assess these similarities [7,8]. In a study done in 1997 on male monozygotic triplets with schizophrenia, MRI findings showed signs of a partially empty sella, and pathological expansion of the suprasellar cistern into the sella turcica was seen in all three children. Moreover, none of the parents showed any signs or symptoms and did not have signs of empty sella on MRI scan [9]. This case concerns with the middle-aged man who was suffering from schizophrenia who reported a rise in violent behavior with no apparent reason. He showed aggressive behavior toward his family and the village members and was a danger to others.

## Case presentation

A 55-year-old male with a well-adjusted premorbid personality, no major medical morbidities, a prior psychiatric history of schizophrenia (diagnosed 20 years ago), a history of poor medication adherence, previously tried on olanzapine, presented to the outpatient clinic of a tertiary care center with acute exacerbation of his symptoms. He presented with acute worsening of irritability, suspiciousness, and aggression for three months and described auditory and tactile hallucinations of someone trying to hold his neck. His wife described a history of disorganized "dangerous behavior" of wandering from home to swim in a dam at 2 am. The symptoms of the patient had aggravated in the last three months, with the advent of tactile hallucinations which led us to suspect organicity.

The patient has been a known case of schizophrenia for the last 20 years and has shown it to three different psychiatrists. He was given three electroconvulsive therapies (ECTs) 20 years ago and was started on olanzapine oral tablets. The patient became noncompliant after two years and again after 10 years, after which he remained compliant on a single dose of olanzapine 20 mg. According to his sister, the patient had a well-adjusted personality before the illness, with a calm demeanor. The patient was admitted under psychiatry with the abovementioned complaints and was started on clozapine 25 mg at night with clonazepam 0.25 mg as needed in case of aggressive behavior. The plan was to increase clozapine to optimal doses and maintain him on the same. 

**Figure 1:**
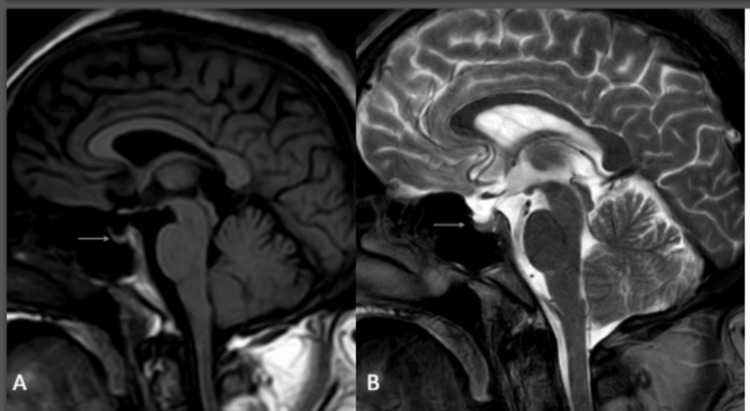
MRI brain sagittal section MRI: Magnetic resonance imaging (A) T1 weighted and (B) T2 weighted showing a partially empty sella (white arrows)

Routine blood investigations were done: complete blood count (CBC), liver function tests (LFT), kidney function test (KFT), and electrocardiogram (ECG), which were within normal limits. During the course of current admission, the patient was newly diagnosed as a case of type 2 diabetes mellitus, and thus olanzapine was omitted in view of metabolic side effects. On general physical examination, the patient was overweight. His vitals were normal, and his central nervous system examination was unremarkable. The patient’s random blood sugar (RBS) was 231 mg/dl (normal value is less than 140 mg/dl), and HbA1C was 7.8 (normal value is 4%-5.6%). Hence, the patient was started on metformin 500 MG twice daily. Clozapine was discontinued due to the risk of metabolic side effects and worsening of diabetes mellitus on clozapine, and the patient was started on risperidone 2 mg once daily. The patient was also noticed to have myoclonic jerks and symptoms of lip-smacking and abnormal rolling movement of the mouth. Tardive dyskinesia was suspected, and an electroencephalogram (EEG) was done to rule out any seizure activity, which was found to be normal. MRI of the brain was done due to suspicion of organicity, which revealed a partially empty sella (Figure 1A-1B).

Over the course of admission, the dose of risperidone was optimized to 4 mg daily and trihexyphenidyl 2 mg daily was added for extrapyramidal side effects. This resulted in an improvement in his aggressive and disorganized behavior and partial reduction in the frequency of auditory hallucinations. Due to concerns about poor medication adherence, injection flupentixol decanoate 20 mg was administered intramuscularly. The patient followed up one month later, where his disorganized behavior had settled considerably and denied any further hearing of voices. The patient was adherent to risperidone, with RBS levels within the normal range.

## Discussion

The pituitary gland is within the sella turcica or the hypophyseal fossa. This structure is present near the center at the base of the cranium and is fibro-osseous. Sella turcica is a concave indentation in the sphenoid bone. Partially empty sella syndrome (ESS) involves the sella turcica being partially empty or filled with cerebrospinal fluid. The zone filled with cerebrospinal fluid that extends through the subarachnoid space to the sella turcica is called the "empty sella" in radiography. There are two kinds of empty sella: the primary empty sella and the secondary empty sella, resulting from a pituitary gland degenerative condition [10]. Mental illnesses such as anxiety or dysthymia with behavioral disturbances were observed quite often (in 80.2% of the 71 cases evaluated) in an investigation of the most common clinical presentations of empty sella [11]. There was only one case report of male monozygotic triplets with schizophrenia who developed the illness at age 20 and had empty sella on brain MRI with extra DNA strands on chromosome 15p [12]. Other reviews showed progressive brain changes in schizophrenia, but there was no evidence of a connection between schizophrenia and empty sella in the literature [13,14]. This chromosomal change might be the cause of any of the two illnesses [15]. Though additional bands of 15p chromosome were implicated as the causative factor for ESS and schizophrenia, many studies have not found this association. In the other study conducted by Wix-Ramos, it was discovered schizophrenia and empty sella in their patient, nobody of his parents had these conditions, nor did any of them have an additional band on the chromosome 15p or any other altercations in the karyotype [16].

In a study which was undertaken by Jönsson among a set of schizophrenic male monozygotic triplets aged 20 years and within the time duration of eight months, the three men showed signs of acute fulminant schizophrenic disorders (DSM-III-R) with auditory hallucinations, bizarre delusions, and thought disturbances [12]. Neuropsychological evaluation revealed comparable, significant declines in executive and attentional skills. Comparable borderline ventricular enlargement was seen on magnetic resonance imaging and expanded subarachnoid spaces across the frontoparietal and basal areas and surrounding the pituitary gland (empty sella). Each boy also exhibited a significant decrease in the ossicular bones on the right side, along with a right-sided hearing impairment. The absence of a documented family history of the illness and the mother's potential prenatal influenza infection were potential etiological factors. It is hypothesized that the very comparable schizophrenia phenotypes were caused by a specific time-programmed sequence of events that were set in motion by a DNA aberration that existed or occurred at conception. An exogenous agent, a spontaneous occurrence, or a recessive mechanism might have caused such an abnormality. Such a DNA modification may be the cause of the psychoses, the decreases in neuropsychological functions, the morphological MRI abnormalities, and the reductions in right-sided ossicles [9]. In the above case study, a patient diagnosed with schizophrenia had an incidental finding of partially empty sella. In patients with ESS, the pituitary gland flattens or decreases, causing the sella turcica to fill with cerebrospinal fluid during imaging rather than the typical pituitary [17]. The most prevalent functional psychotic condition is schizophrenia, which can show in a wide range of ways in its sufferers. Although there is high heredity associated with schizophrenia, little is known about the disorder's etiology [18].

## Conclusions

In individuals diagnosed with schizophrenia, the identification of an empty sella turcica on radiological imaging can sometimes occur as an incidental observation. While seemingly unrelated, there is emerging speculation within the medical community regarding a potential shared genetic underpinning between schizophrenia and the presence of an empty sella turcica. The suggestion of a shared chromosomal mutation linking schizophrenia and empty sella turcica stems from ongoing genetic research aimed at unraveling the intricate molecular pathways involved in psychiatric disorders. Investigations into the genetic basis of structural brain abnormalities, such as the empty sella turcica, have provided additional insights into potential genetic overlaps with psychiatric conditions. One hypothesis posits that certain genetic mutations or polymorphisms may predispose individuals to both schizophrenia and structural brain abnormalities, such as alterations in pituitary gland development or morphology. These genetic aberrations could disrupt normal neurodevelopmental processes, leading to aberrant brain structure and function, ultimately manifesting as psychiatric symptoms characteristic of schizophrenia. Despite these intriguing conjectures, it is essential to approach the association between schizophrenia and empty sella turcica with caution, as the available evidence remains preliminary and inconclusive. Further interdisciplinary research integrating genetics, neuroimaging, and clinical psychiatry is required to elucidate a link between the two. Such insights hold the potential to advance our understanding of the pathophysiology of schizophrenia and pave the way for more targeted diagnostic and therapeutic interventions tailored to individual genetic profiles.
